# Multiple *Trypanosoma *infections are common amongst *Glossina *species in the new farming areas of Rufiji district, Tanzania

**DOI:** 10.1186/1756-3305-4-217

**Published:** 2011-11-17

**Authors:** Imna I Malele, Henry B Magwisha, Hamisi S Nyingilili, Kamilius A Mamiro, Elipidius J Rukambile, Joyce W Daffa, Eugene A Lyaruu, Lupakisyo A Kapange, Gideon K Kasilagila, Nicodemus K Lwitiko, Halifa M Msami, Elikira N Kimbita

**Affiliations:** 1 Tsetse & Trypanosomiasis Research Institute (TTRI), Box 1026 Tanga, Tanzania; 2 Central Veterinary Laboratory (CVL), Box 9254 Dar Es Salaam, Tanzania; 3 Ministry of Livestock Development & Fisheries (MLDF), Box 9152, Dar Es Salaam, Tanzania; 4 District Veterinary Office, Rufiji, Tanzania; 5 Sokoine University of Agriculture (SUA), Box 3019, Morogoro, Tanzania

**Keywords:** tsetse, *Trypanosoma*, *Glossina*, traps, attractants, livestock, trypanosomiasis, pastoralists, Tanzania

## Abstract

**Background:**

Tsetse flies and trypanosomiasis are among several factors that constrain livestock development in Tanzania. Over the years Rufiji District was excluded from livestock production owing to tsetse fly infestation, however, a few years ago there was an influx of livestock following evictions aimed at conserving the Usangu wetlands.

**Methods:**

A study was conducted to determine the efficiency of available traps for catching tsetse flies, *Glossina *species infesting the area, their infection rates and *Trypanosoma *species circulating in the area. Trapping was conducted during the semi dry season for a total of 30 days (ten days each month) during the onset of the dry season of May - July 2009. Harvested flies after every 24 hours were dissected and examined under a light microscope for trypanosome infections and whole fly DNA was extracted from 82 flies and analyzed for trypanosomes by polymerase chain reaction (PCR) using different sets of primers.

**Results:**

The proportions of total tsetse catches per trap were in the following decreasing order S3 (33%), H-Trap (27%), Pyramidal (19%), sticky panel (11%) and biconical trap (10%). Of the 1200 trapped flies, 75.6% were identified as *Glossina pallidipes*, 11.7% *as G. brevipalpis*, 9.6% as *G. austeni *and 3.0% *G. morsitans morsitans*. Dissections revealed the overall infection rate of 6.6% (13/197). Whole DNA was extracted from 82 tsetse flies and the prevalence of trypanosomes circulating in the area in descending order was 92.7% (76/82) for *T. simiae*; 70.7% (58/82) for *T. brucei *types; 48.8% (40/82) for the *T. vivax *types and 32.9% (27/82) for the *T. congolense *types as determined by PCR. All trypanosome types were found in all tsetse species analysed except for the *T. congolense *types, which were absent in *G. m. morsitans*. None of the *T. brucei *positive samples contained human infective trypanosomes by SRA - PCR test

**Conclusion:**

All tsetse species found in Rufiji are biologically important in the transmission of animal trypanosomiasis and the absence of *T. congolense *in *G. m. morsitans *could be a matter of chance only. Therefore, plans for control should consider all tsetse species.

## Background

Tsetse flies and trypanosomiasis are a serious constraint to livestock sector development in sub Saharan Africa. The disease lowers productivity in livestock, reduces cattle density up to 70%, sale of meat and milk by 50% and calving rates by 20% and calf mortality by 20% [1]. In Tanzania, tsetse-borne diseases, and in particular bovine trypanosomiasis, which is one of the two most important diseases that are responsible for reduced livestock productivity and together with tick-borne diseases they are responsible for 75% of the morbidities and mortalities in cattle [2].

Southern Tanzania is one of the areas in the country that are tsetse infested and keeping of livestock has been severely constrained by tsetse transmitted trypanosomiasis [3]. In early 2007, the country saw a rapid increase in the number of livestock settled in Rufiji district, of the Coastal Region of Tanzania, following the evacuation of livestock from Usangu and Ihefu areas which were declared conservation areas and are the key water sources for hydroelectric power generation [4]. Many pastoralists opted to settle in the Coastal Region, which has a low human population density hence ensures ample grazing land for their animals. Settlement of pastoralists and their animals in the district started a long time ago following pressures associated with land use in the northern circuit of the country [5]. However, animal populations in Rufiji district increased rapidly as a result of this eviction from the wetland sources from 20,000 livestock in 2005/2006 to about 140,000 by mid 2008/2009.

Settlement of these animals came without much preparation to avert the problem caused by tsetse and trypanosomiasis in the pasture areas. There was no baseline information on the situation of tsetse and trypanosomiasis; so despite the availability of plenty of grazing areas for animals, the pastoralists were confronted by a serious challenge of tsetse and trypanosomiasis, which has become a major stumbling block to livestock sector development in Rufiji. Records on tsetse and trypanosomiasis in southern Tanzania are very scant and the available records are by Connor and Halliwell [6] and MacLennan [3], who reported on bovine trypanosomiasis and tsetse distribution in Mtwara region respectively, an area bordering Mozambique. Prior to this study, there was no information regarding tsetse and trypanosomiasis in the Rufiji district.

This study was therefore conducted in order to investigate the magnitude of tsetse and trypanosomiasis distribution through investigation of tsetse species, densities, infection rates, trypanosome types circulating in the area in order to determine the magnitude of tsetse and trypanosomiasis (T & T) for the purpose of planning control interventions against the vector and trypanosomiasis for improved livestock production in the District. The information gathered will contribute towards planning a control intervention against T & T not only in Rufiji and the Coastal regions, but in southern Tanzania as a whole; against African Animal Trypanosomiasis (AAT) and Human African Trypanosomiasis (HAT) by bearing in mind that Rufiji is at the periphery of the southern silent foci of sleeping sickness, Nachingwea [7]. Control of T & T will significantly improve agricultural activities and human health in the area.

## Materials and methods

### Study site

Rufiji district located at S-8.1022° latitude and E 38.3756° longitude; is one of the districts in the Coastal Region and offers potential for agricultural and livestock production. The district covers 39.8 percent of the total 33,539 km^2 ^of Coastal region area, which offers potential for mixed agriculture [8]. The average rainfall in this zone ranges from 900 mm - 1000 mm per year. Formerly, out of the total countrywide population of livestock, only 0.44% of the cattle and 1.7% of the sheep and goat populations were available in the area before the mass settlement in 2007 [8]. According to the 2002 national census, Rufiji had about 22.8% (202,001) of the total population of the Coastal region, which stood at 885,017. The district is also endowed with 482,466 potential arable hectares; however, only 20.7% is utilized for crop production. Residents practice subsistence farming of crops like cassava, rice, maize, sweet potatoes and legumes, sorghum, cashew nuts and coconuts and fruits of various types especially oranges and mangoes. Small scale fishing is practiced in the Indian Ocean and Rufiji River [8]. The influx of pastoralists from the northern areas of the country is set to increase agro pastoral practices in the district.

### Entomological surveys

#### Trap deployment

For easy implementation of the study, the district was divided into three blocks (A, B and C) as follows:

##### Block A - covering Mkongo Division, Utete Ward

Traps were deployed in Siasa Village, Utete West, situated on the western part of the district adjacent to the wildlife management area bordering Selous Game Reserve.

##### Block B - Kikale Division, Mtunda Ward

Traps were deployed in Muyuyu village near Mtunda forest. This ward is situated on the northern part of Rufiji River.

##### Block C - Covering Muhoro Division, Chumbi Ward

**Figure 1:**
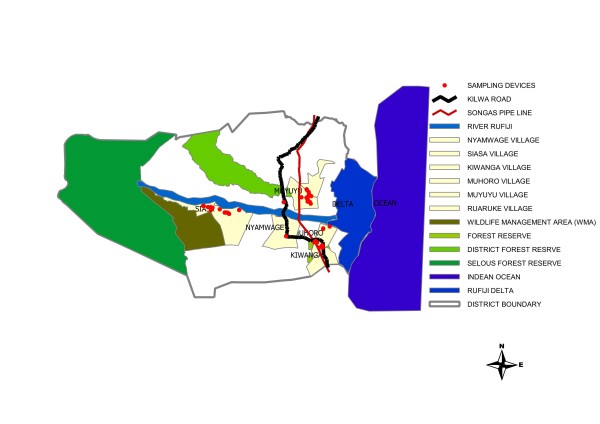
**Tsetse surveyed areas in Rufiji District**.

Traps were deployed in Kiwanga and Chumbi villages in the southern part of the district (Figure 1).

### Trapping of tsetse flies

Information about the study area was obtained from the District Veterinary personnel before initiating the study. Having identified the tsetse infested areas, a total of 15 traps were deployed in selected points along transects in order to cover all different vegetations that were found in the three blocks. These included conventional stationary traps namely Biconical [9], Pyramidal [10], S3 [11], H-trap [12] and sticky panels [13]. In each block, five different traps were used and these were deployed at 10.00 am every morning and emptied at the same time the next day. They were placed 150 - 200 metres apart depending on the vegetation cover of the area surveyed. All traps were baited with acetone, released from a 6 mm diameter hole in the top lid of a half litre plastic container. Study areas and trap locations were geo-referenced by a Global Positioning System (GPS). The trapped flies were collected and sorted into respective sexes, species and numbers. Trapping was conducted during the semi dry season for a total of 30 days (ten days each month) during the onset of the dry season i.e. from May to July 2009.

#### Determination of tsetse species, infection rates and trypanosome types circulating in the area

Trapped tsetse were harvested after every 24 hrs, counted and sorted into respective species and sexes. The flies were then dissected and organs examined under a light microscope to determine the presence of trypanosomes. The flies were dissected in physiological buffered saline (PBS) using a stereo dissection microscope and target tissue (mouth parts, salivary gland and midguts) and fly carcasses were placed in 100% ethanol for storage at -20°C and later DNA extraction. DNA from whole flies from 82 tsetse flies were also extracted using a Qiagen Kit for trypanosome characterization as previously described [14] showing that trypanosomes can be analysed from DNA extracted from whole flies without dissection. Amplification using Phusion Taq (FinnEnzyme) for different species-specific primers and test primers in a DNA thermal cycler (Applied Biosystem) in a final volume of 25 μl was carried out following published protocols and amplification conditions as indicated in Table 1.

**Table 1:** Trypanosome species specific Primers used in this study

Primer Code	Sequence	Base pairs (bp)	Reference
TV	3-ACTCAAAATCGTGCACCTCG-5 5-CCCGGCAGGTTGGCCGCCATC-3	399	[21]

TCK	5-GTG CCC AAA TTT GAA GTG AT-3 5-ACT CAA AAT CGT GCA CCT CG-3	294	[22]

TCF	5-GGA CAC GCC AGA AGG TAC TT-3 5-GTT CTC GCA CCA AAT CCA AC-3	350	[22]

TCS	5-CGA GAA CGG GCA CTT TGC GA-3 5-GGA CAA AGA AAT CCC GCA CA-3	316	[22]

TBR	5-CGAATGAATATTAAACAATGCGCAG-3 5-AGAACCATTTATTAGCTTTGTTGC-3	177	[23]

TSM	5-CCGGTCAAAAACGCATT-3 5-AGTCGCCCGGAGTCGAT-3	437	[22]

SRA A & E	A (5-GACAACAAGTACCTTGGCGC-3) E (5-TACTGTTGTTGTACCGCCGC-3)	460	[24]

*Trypanosoma spp *(ITS 1 CF & ITS 1 BR)	5'-CCGGAAGTTCACCGATATTG-3' 5'-TTGCTGCGTTCTTCAACGAA-3'	Species-specific sizes	[25]

Internal transcribed spacer (ITS) amplification was carried out in 20 μl reaction mixture containing 11.4 μl distilled water, 4 μl of 5× Phusion HF buffer (FinnEnzyme), 0.4 μl 10 mM dNTPs, 1 μl for each Primer, 0.2 ul Phusion DNA polymerase and 2 μl DNA template except for screening the presence of serum associate (SRA) gene, where 4 μl of template was used. The polymerase chain reaction (PCR) condition involved an initial denaturation step at 98°C for 30 second, followed by 30 cycles of 98°C for 30 s, 60°C for 40 s, 72°C for 30 s with a final elongation step at 72°C for 5 min. 5 μl of PCR product was mixed with standard loading dye (Hyperladder) and electrophoresed in 1.5% agarose stained with ethidium bromide (5 μg/ml) and the product visualized and photographed under ultraviolet illumination. A positive control (with reference genomic DNA) and negative control (without DNA, only with distilled water) were included in each set of reactions.

## Results

### Entomology surveys: Tsetse composition and distribution

A total of 1200 flies were trapped during this study and four tsetse species were identified as *Glossina pallidipes, G. brevipalpis, G. m. morsitans *and *G. austeni*. The proportional abundance of all trapped flies were 75.6% (908) for *G. pallidipes*, 11.7% (140) for *G. brevipalpis*, 9.7% (116) for *G. austeni *and 3.0% (36) *G. m. morsitans*. Flies trapped in all blocks/villages sampled are indicated in Table 2.

**Table 2:** Tsetse species and apparent densities in the surveyed areas of Rufiji district

Blocks	Division	Ward	Village	Tsetse species	Overall Total flies caught	Overall Apparent density (Flies/trap/day)
A	Mkongo	Utete	Siasa	*G. pallidipes* *G. brevipalpis* *G. austeni* *G. m. morsitans*	400 (154 males, 246 females)	0.90

B	Kikale	Mtunda	Muyuyu	*G. pallidipes* *G. brevipalpis* *G. austeni*	99 (10 males, 89 females)	0.22

C	Muhoro	Chumbi	Kiwanga near Kiwanga forest.	*G. pallidipes* *G. brevipalpis* *G. austeni*	700 (200 males, 500 females)	1.60

### Tsetse fly response to traps

**Figure 2:**
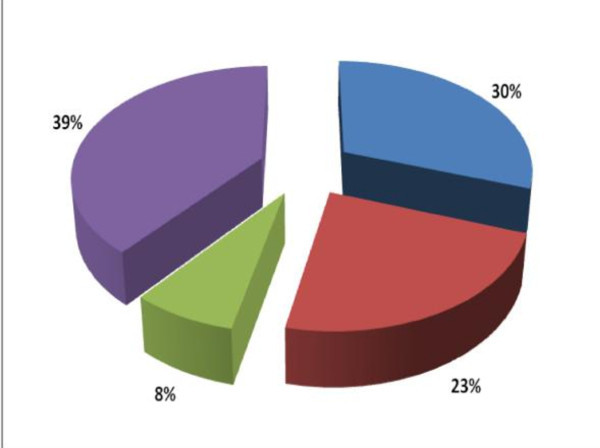
**Proportion of trypanosomes types circulating in Rufiji. **. Trypanosoma simiae - 39%. T. brucei brucei - 30%. T. vivax- 8%. T. congolense - 23%. T. congolense includes T. congolense savannah, T. congolense kilifi, and T. congolense forest.

S3 and H traps caught more flies than other traps (Figure 2). *G. pallidipes *was found in all traps used in this study, whereas *G. m. morsitans *was trapped by H-Trap and Sticky panels. *G. austeni *was trapped by S3, H-Trap and Sticky panels. In addition, *G. brevipalpis *were found trapped by H and Pyramidal traps.

### Infection rates in tsetse

#### (i) Examination of dissected tsetse flies by microscopy

A total of 197 *G. pallidipes *were dissected and examined microscopically for trypanosomes and the overall infection rate ranged from 2.5% in midguts and 6.6% in proboscides and most of the infecting trypanosomes were *T. vivax *and *T. congolense*. The results are presented in Table 3.

**Table 3:** Microscopic infection rates in tsetse

Tsetse species	Infected organ	Trypanosome spp/type	% infection
*G. pallidipes*	proboscides	*T. vivax*	13 (6.6%)

	midgut	*T. brucei*	5 (2.5%)

	midgut	*T. congolense*	5 (2.5%)

#### (ii) Molecular characterization of trypanosomes

**Figure 3:**
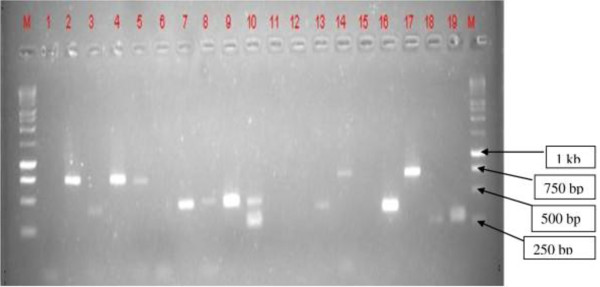
**Trypanosome DNA products by ITS - PCR from Rufiji tsetse samples**. Numbers 4, 5, 14, and 17 are *T. congolense *savannah ~700 bp. Numbers 3,7,8,9,10,13,16 are *T. simiae *~ 400 bp. Numbers 10 and 19 are *T. savannah Tsavo *~ 370 bp. Number 18 is *T. vivax~ *250 bp. Number No. 2 is positive control for *T. congolense *savannah. Number 1 is negative control. M = 1 kb DNA ladder.

**Figure 4:**
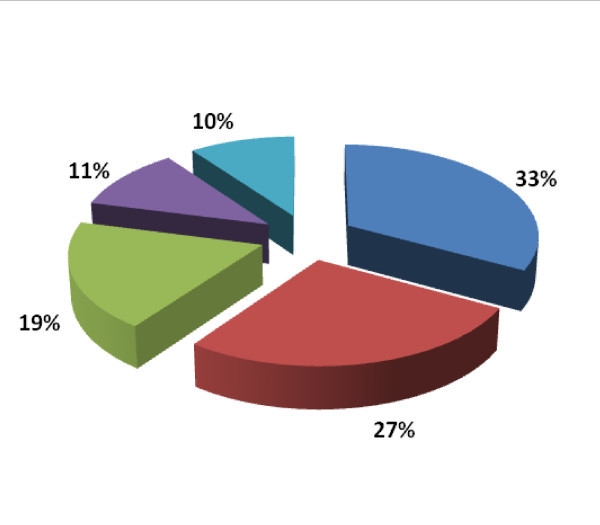
**Total tsetse catches per trap type. **S3 - 33%. H-Trap - 27%. Pyramidal - 19%. Sticky panel - 11%. Biconical - 10%.

Results of DNA from 82 flies analyzed for trypanosomes by PCR are shown in Table 4. Initially, the intention was to determine trypanosome infection by ITS - PCR (Figure 3); however due to high prevalence of mixed infection it was decided to use species specific primers in order to identify the exact trypanosome species circulating in the area; also by bearing in mind that this was the first time molecular characterization of trypanosomes circulating in tsetse from this area was carried out (Table 4 & Table 5). None of the *T. brucei *positive samples contained human infective trypanosomes by SRA - PCR test. The proportions of trypanosome types circulating in the area are presented in Figure 4.

**Table 4:** Trypanosomes infection rates per tsetse species

Tsetse spp	Tb	TC*	Tv	Tsm
*G. pallidipes*	45/82 (54.9%)	19/82 (23.2%)	26/82 (31.7%)	58/82 (70.7%)

*G. m. morsitans*	3/82 (3.7%)	0	3/82 (3.7%)	4/82 (4.9%)

*G. brevipalpis*	4/82 (4.9%)	3/82 (3.7%)	4/82 (4.9%)	7/82 (8.54%)

*G. austeni*	6/82 (7.3%)	5/82 (6.1%)	7/82 (8.54%)	7/82 (8.54%)

**Overall**	**58/82 (70.73%)**	**27/82 (32.93%)**	**40/82 (48.78%)**	**76/82 (92.7%)**

* Includes the total genotypes of *T. congolense. Tb = T. brucei; TC = T. congolense; Tv = T. vivax; and Tsm = T. simiae Tsavo and T. congolense Tsavo*

**Table 5:** The prevalence of *T.congolense *genotypes per tsetse species

Tsetse spp	Tck	Tcf	Tcs
*G. pallidipes*	7/82 (8.5%)	1/82 (1.22%)	11/82 (13.4%)

*G. m. morsitans*	0/82	0/82	0/82

*G. brevipalpis*	2/82 (2.4%)	0/82	1/82 (1.22%)

*G. austeni*	4/82 (4.9%)	0/82	1/82 (1.22%)

**Overall**	**13/82 (15.85%)**	**1/82 (1.22%)**	**13/82 (15.85%)**

NB: *Tck = T. congolense kilifi; Tcf = T. congolense forest; and Tcs = T. congolense savannah*

### The *T. congolense genotypes*

The three T. congolense genotypes were found circulating in the area as indicated in Table 5.

### Pattern of Mixed infection of trypanosomes

The pattern of mixed infection and occurrence was 13.1% (11/82) for single infection; 37.8 (31/82) for double; 35.4% (29/82) for triple infection. Those which occurred in a mixture of four together were 9.8% (8/82). Only one (1.2%) set of infection had a mixture of five different types of trypanosomes.

The proportion of analysed samples from each block was 19.5% (16) for Utete (Block A); 24.4% (20) for block B (Muyuyu); 56.1% (46) for block C (Kiwanga and Muhoro).

## Discussion and conclusion

The study found that the area was infested by four species of tsetse, namely *G. pallidipes*, *G. m. morsitans*, *G. brevipalpis *and *G. austeni*. Individual tsetse species responded differently to the traps used in the study. *G. pallidipes *were found in all traps namely Biconical, Pyramidal, S3, H-trap and Sticky panel. *G. m. morsitans *were found in H-trap and Sticky panel; *G. austeni *were found in S3, H-trap and sticky panel whereas *G. brevipalpis *were also found in Pyramidal and H-trap. The apparent density obtained was not very high compared to flies seen visiting most traps. This could be due to the reluctance of most *Morsitans *tsetse flies to enter into stationary traps. Observation made by Shaw *et al*. [15] on *G. pallidipes *and *G. morsitans *indicated that only 47 - 30% of tsetse approaching a trap landed on it or entered it, which means that about 53 - 70% of tsetse visiting the trap did not contact it. This is the phenomenon that was also noticed in Rufiji. Flies would land on traps without entering the retaining cage.

About five species of trypanosomes, the causative parasites of AAT in livestock were found circulating in tsetse. We used species specific primers in order to obtain the exact types of species found in the area. However, we did not use the *T. godfreyi *primers, or the *T. vivax *specific primers for *T. vivax *western types, which could have affected the *T. vivax *prevalence in the area and the total number of trypanosomes circulating in the area. So far tsetse flies found in Rufiji District are infected by *T. vivax, T. simiae*, *T. brucei *types and the three genotypes of *T. congolense *(*T. congolense savannah, T. congolense forest and T. congolense kilifi)*. *T. simiae *included the *T. simiae Tsavo *and *T. congolense Tsavo *[16].

The *T. congolense *types were pooled together to give the total *congolense *types in the area (Table 4); however, when analysed individually, *G. m. morsitans *were not infected by the *T. congolense *parasite. *Trypanosoma congolense *kilifi was found in *G. pallidipes *(8.5%), *G. brevipalpis *(2.4%) and *G. austeni *(4.9%). *Trypanosoma congolense *forest was only found in *G. pallidipes *(1.2%). *Trypanosoma congolense *savannah was found in all tsetse species analysed except in *G. m. morsitans*. None of the *T. brucei *positive samples were SRA - PCR positive. It was important to have a confirmatory test of the infective status of *T. brucei *positive samples because Rufiji is at the periphery of the southern silent sleeping sickness foci (Nachingwea). Also with movements of animals from northern and western parts of Tanzania through tsetse infested HAT active foci; the likelihood of human infective trypanosomes being introduced in Rufiji cannot be ignored. In areas like Uganda, livestock movements have been behind the spread of HAT in the country [17].

The results have indicated the importance of *G. austeni *and *G. brevipalpis *in the epidemiology of animal trypanosomiasis through harboring animal infective parasites. It was also interesting to note the presence of *T. brucei *and *T. vivax *infections in G. *brevipalpis *as well as *G. austeni*. Available documented reports tend to link *G. austeni *and *G. brevipalpis *with Suidae related trypanosomes which are mainly *T. simiae *due to its feeding preferences of warthogs and hippopotamus [18], respectively. However, in this study, the two tsetse species (*G. brevipalpis and G. austeni*) were found to be equally infected with all trypanosome types circulating in the area more or less like the *G. pallidipes *and *G. m. morsitans *species which are known to play a major role in the epidemiology of AAT. The high prevalence of *T. simiae *in the area gives the indication that pig production in Rufiji District could be adversely affected or rather impossible.

### Pattern of trypanosome infections

The common single infections were the *T. simiae *with total occurrence of 8, six in *G. pallidipes *and one each in *G. brevipalpis *and *G. m. morsitans *and none in *G. austeni*. The least common single infection pattern was the *T. vivax*. The *T. congolense *forest (Tcf) was found once in *G. pallidipes *only and occurred in a mixture of five infections (Tb + Tcf + Tcs + Tv + Tsm). The common pattern of triple infection was Tb + Tv + Tsm with a total 19 scores, highest in *G. pallidipes *(14/19), followed in *G. m. morsitans *(3/19) and one each in *G. austeni *and *G. brevipalpis*. The common pattern of double infections was for Tb and Tsm with a total of 22, highest in *G. pallidipes *(20/22); one each in *G. brevipalpis *and *G. austeni*. Other double infections were for Tv and Tsm with total occurrences of 6, three in G. *pallidipes*; 2 in *G. brevipalpis *and 1 in *G. austeni*. Other patterns were of Tb + Tv + Tcs recorded in *G. austeni *only; whereas the infection of Tb + Tck + Tcs + Tsm was recorded in *G. brevipalpis *only. The occurrence of four different infections of trypanosome in *G. brevipalpis *confirms earlier findings of Namabolo *et al*. [19], on the importance and role of *G. brevipalpis *and *G. austeni *in the epidemiology of AAT in tsetse infested areas in South Africa. Lastly there were three scores of Tb + Tck + Tv + Tsm all recorded in *G. austeni *only (Table 6).

**Table 6:** Trypanosome mixed infection patterns and their occurrence in tsetse

Number of trypanosome species	Pattern of trypanosome infection	*G. austeni*	*G. brevipalpis*	*G. m. morsitans*	*G. pallidipes*	Total	% prevalence
2	Tv + Tsm	1	2		3	6	7.32

5	Tb+ Tcf+ Tcs + Tv + Tsm				1	1	1.22

4	Tv+Tck+Tcs+Tsm				1	1	1.22

3	Tb+Tcs+Tsm				3	3	3.66

2	Tck + Tsm				3	3	3.66

4	Tb+Tv+Tcs+Tsm				2	2	2.44

3	Tb+Tck+Tsm				2	2	2.44

3	Tv+Tcs+Tsm				2	2	2.44

3	Tck+Tcs+Tsm				1	1	1.22

3	Tb+Tv+Tsm	1	1	3	14	19	23.17

3	Tck+Tv+Tsm	1			1	1	1.22

2	Tb+Tsm	1	1		20	22	26.83

1	Tsm		1	1	6	8	9.76

1	Tb				4	4	4.88

1	Tv				1	1	1.22

3	Tb+Tv+Tcs	1				1	1.22

4	Tb+Tck+Tcs+Tsm		1			1	1.22

4	Tb+Tck+Tv+Tsm	3	1			4	4.88

		**8**	**7**	**4**	**64**	**82**	**100.02**

Tb - T. brucei; Tcs - T. congolense savannah; Tcf - T. congolense forest; Tck - T. congolense kilifi; Tv - T. vivax; Tsm - T. simiae Tsavo and T. congolense Tsavo

In this study we recorded the presence of the three distinct subspecies of *T. congolense*, which are known to have various severity/virulence in cattle, which is an indication of how important is AAT in the area. The western part of the district (Utete) borders the Selous game reserve, which could be a source of a cocktail of trypanosomes in tsetse as a result of close interactions between livestock and wild animals in the area. It is possible that tsetse flies in this area obtain blood meals from both wild and domestic animals, and this would increase the trypanosomes types found in the vector.

Mixed infections of trypanosomes seem to be a usual phenomenon in southern Tanzania. Connor & Halliwell [6] reported 93 positive cattle with trypanosome of which 56% were infected by *T. congolense*; 17% by *T. vivax *and 2.2% with *T. brucei*. Five animals had mixed infections of the three groups of trypanosomes (Tc, Tb & Tv). This points towards the severity of AAT in cattle in southern Tanzania and this exacerbated animal losses incurred by pastoralists when they moved to southern Tanzania [4] such that deliberate efforts need to be in place to control tsetse in order to break the transmission cycle to domestic animals and thus improve livestock production and productivity in the area.

AAT risk is usually linked to the density of the vector and the trypanosome infection rates and from this area both parameters exists [20] i.e. tsetse and high trypanosome infection rates. Each tsetse species found in the area is significantly important due to the fact that they were all more or less found infected by all types of trypanosomes circulating in the area. *T. vivax *and *T. congolense *are regarded as major pathogens of cattle and other ruminants, while *T. simiae *causes high mortality in domestic pigs. The trypanosome types of *T. vivax, T. brucei *and *T. simiae *were found in all four tsetse species found in the area; on the other hand *T. congolense *types were not found in *G. m. morsitans*. This is an indication that the dominance of *G. pallidipes *should not exclude other tsetse species when planning for tsetse control strategies. Kiwanga village which is near Kiwanga forest (Block C) was relatively undisturbed when we sampled, however, pastoralists had started settling in search of pasture for their animals and this could explain the relatively high density of flies compared to other blocks. Block B was affected by the settlement of pastoralists, as by the time of sampling much tree felling was underway to clear areas for farming and other daily related activities; whereas in block A (Utete) we were able to trap flies in the first ten days of May. In June the area was burnt, hence we couldn't trap flies despite the fact that the area is near the Selous game reserve where the fly density is high. The different types of trypanosomes found in tsetse directly implicates tsetse in the transmission of AAT in livestock; and this is the first study to show the role of tsetse species in the transmission of AAT in southern Tanzania.

## Competing interests

The authors declare that they have no competing interests.

## Authors' contributions

IIM, HBM, KAM, EJR, HMM and ENK conceived the study, designed and coordinated the study; IIM, HBM, ENK performed data analysis and drafted the manuscript. KAM, JWD, EAL, GKK, LAK and NKL participated in the field work. HSN, EAL, IIM and LAK carried out the laboratory analysis on collected samples. EAL, HSN drew the map of the sampling sites. All authors read and approved the final version of the manuscript.
